# Supplementary material for: Research on intelligent routing with VAE-GAN in the internet of body
Source: PLoS One. 2025 Feb 10;20(2):e0317698. doi: 10.1371/journal.pone.0317698 (PMC11809925; doi:10.1371/journal.pone.0317698)
Supplement: Attachment — Submitted filename: Response to Reviewers.docx [file pone.0317698.s001.docx]

Response to Reviewers

**Dear Reviewers,**

**Thank you very much for your review. We have carefully revised and improved according to your suggestions. Please refer to the details below. We hope to meet the requirements of the journal. If you have any other questions, please contact us.**

**Wishing you a happy life and good health.**

**The authors**

**These are all Response.** （All responses from the authors are presented with a gray background.）

**Please use the space provided to explain your answers to the questions above. You may also include additional comments for the author, including concerns about dual publication, research ethics, or publication ethics. (Please upload your review as an attachment if it exceeds 20,000 characters)**

**Reviewer #1: In this paper the authors proposed an artificial intelligence routing algorithm, combines the variational autoencoder (VAE) and the generative adversarial network model (GAN) to construct a VAE-GAN model to generate multiple sets of data to achieve data enhancement in the Internet of Body. The optimization goals are to maximize the throughput of the Internet of Body and minimize the transmission cost. Here are my comments:
1. Title of the paper doesn’t reflect the body or contribution of the work. Title should be revised.**

1.Author's response: Thank you for the review. The title has been updated. The new title is *Research on Intelligent Routing with VAE-GAN in the Internet of Body.*

**2. Paper has some merit but it is not well written. Very poor type setting.**

2.Author's response: Thank you for the review. We have made detailed modifications, please see *Revised Manuscript with Track Changes*. **3. Introduction and related work is incomplete and no flow in the paper. It’s really difficult to read the paper.**

3.Author's response: Thank you for the review. We have made new modifications. And added some paper to *RELATED WORK*. And Revised the grammar of the entire text.

**4. Even through many recent works in WBAN domain the authors provide only 13 references and most of them are old.**

4.Author's response: Thank you for the review. We modified and added more paper in *REFERENCES*.

**5. Various modeling like channel model, network model is missing in the paper.**

5.Author's response: Thank you for the review. The network model is in manuscript lines 202 – 239.

**6. Algo. 1 is confusing and it did not reflect well in the body of the paper.**

6.Author's response: Thank you for the review. Figure 1 is to illustrate the application scenario of this manuscript, which is to solve the routing problem of Internet of Body during large-scale access, and to address the usage scenario of h Internet of Body. **7. Simulation parameters are not provided in the paper. Only 3 outputs can not justify the novelty of the work.**

7.Author's response: Thank you for the review. We added more simulation parameters and experiments in this manuscript. **8. I suggested to the author to read recent articles and add them in related works. Some suggestion: 10.1109/JSEN.2024.3440412, https://doi.org/10.1016/j.iot.2024.101151, 10.1109/ACCESS.2023.3236403, 10.1109/TCE.2024.3412942, 10.1109/JIOT.2024.3458976, 10.1109/ACCESS.2024.3476424**

8.Author's response: Thank you for the review. We modified and added more paper (including the above papers) in *REFERENCES*.

**Reviewer #2: I think the article is excellent, I congratulate the authors. Some suggestions could be considered:
1. Algorithm specificity: Explaining more clearly why VAE and GAN were combined instead of other approaches could add value.**

1.Author's response: Thank you for the review. VAE and GAN mainly solve the problem of limited high-value samples, which has been added to the manuscript. **2. Results and conclusions: While it is mentioned that the experiments demonstrated good results, it would be useful to briefly indicate what type of improvement or specific metric was achieved (e.g., latency reduction, increased network efficiency, lower error rate, etc.).**

2.Author's response: Thank you for the review. Parameter analysis has been added in the experiment. Like: The normalized throughput tends to stabilize, indicating that the model can apply the influence of high dynamics.

**3. Although I do not see it as 100% necessary, I think it would be great to incorporate a comparison with fuzzy logic. This could significantly strengthen the work; I recommend it for the following reasons:
1. Combination with AI: Fuzzy logic is not incompatible with artificial intelligence techniques such as neural networks, GANs, or VAE. In fact, one can explore how systems based on fuzzy logic can complement or be combined with generative models, to handle additional uncertainties in the data generation or route prediction process. 2. Increased Interpretability: One of the problems with AI-based models, such as GANs, is the “black box” they are based on, which makes it difficult to interpret their decisions. Fuzzy logic could offer a way to add interpretability to the model, as routing decisions can be explained in terms of fuzzy rules, such as “If congestion is high and latency is low, then this route is suitable.” This could make your model more understandable and reliable in medical or critical settings.
3. Efficiency and Computational Resources: Fuzzy logic can be computationally lighter than complex models based on deep or generative neural networks, which could be an important advantage if real-time performance and resource efficiency are crucial in the use case (as mentioned in your abstract about the need to use few computational resources).
4. Routing Optimization: Fuzzy systems can be effectively integrated into routing algorithms to optimize network resource usage by dynamically adjusting routing decisions based on context and changing network conditions. You could compare whether fuzzy logic offers an advantage over your VAE-GAN model, especially when dealing with non-deterministic routes and decisions based on multiple imprecise factors.
5. Simplicity and Flexibility: Fuzzy logic allows for simpler and more flexible representation of relationships between variables, without the need for strict, discrete rules. In the case of your work, you could integrate fuzzy logic to handle different degrees of “quality” or “reliability” of routing paths, rather than simply classifying paths as optimal or non-optimal, which could be a more robust approach to varying network conditions.
In summary, how could you integrate this recommendation in your paper: You could include a section in your paper where you briefly describe the advantages of fuzzy logic compared to deep learning-based methodologies (such as VAE-GAN), and discuss how they could complement or compare each other to solve the routing problem in "Internet of the Body" networks.
Additionally, I dare to send you a small idea of a proposal.
"Although the proposed model based on VAE-GAN has shown a significant improvement in real-time route optimization, it is interesting to consider the integration of fuzzy logic as a possible alternative or complement. Fuzzy logic could offer advantages in making more interpretable and robust decisions in the face of uncertain and fluctuating network conditions, such as congestion or variability of sensor data. Furthermore, the simplicity and flexibility of fuzzy logic could reduce computational complexity in situations where resources are limited. Comparing these approaches and exploring their combination could result in even more efficient optimization of routing in the Internet of the Body, especially in dynamic and high-performance scenarios."**
